# Modelling the non-local thermodynamic equilibrium spectra of silylene (SiH_2_)†

**DOI:** 10.1039/d1cp00839k

**Published:** 2021-04-29

**Authors:** Victoria H. J. Clark, Sergei N. Yurchenko

**Affiliations:** Department of Physics and Astronomy, University College London Gower Street WC1E 6BT London UK v.clark.17@ucl.ac.uk s.yurchenko@ucl.ac.uk

## Abstract

This paper sets out a robust methodology for modelling spectra of polyatomic molecules produced in reactive or dissociative environments, with vibrational populations outside local thermal equilibrium (LTE). The methodology is based on accurate, extensive ro-vibrational line lists containing transitions with high vibrational excitations and relies on the detailed ro-vibrational assignments. The developed methodology is applied to model non-LTE IR and visible spectra of silylene (SiH_2_) produced in a decomposition of disilane (Si_2_H_6_), a reaction of technological importance. Two approaches for non-LTE vibrational populations of the product SiH_2_ are introduced: a simplistic 1D approach based on the Harmonic approximation and a full 3D model incorporating accurate vibrational wavefunctions of SiH_2_ computed variationally with the TROVE (Theoretical ROVibrational Energy) program. We show how their non-LTE spectral signatures can be used to trace different reaction channels of molecular dissociations.

## Abstract 2

Vibrational populations of molecules produced in reactions can be very different from the Boltzmann distribution. These so-called non-LTE populations encode information about the structural reaction dynamics and can manifest in the molecular spectra.

## Graphical abstract

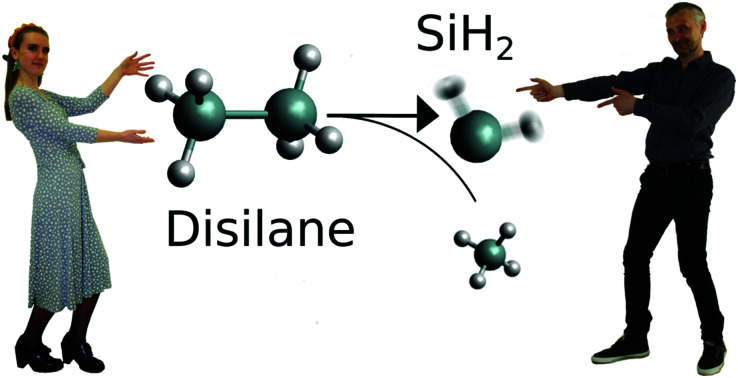

## 1 Introduction

Normally, molecules are assumed to be in local thermal equilibrium pertaining to a given temperature with the internal degrees of freedom (electronic–rotation–vibration) characterized by the Boltzmann distribution. However many different physical chemical, experimental and technological processes produce molecules that do not satisfy the Boltzmann law and as consequence have unusual, non-local thermal equilibrium (non-LTE) spectroscopic signatures. Molecules produced in reactions do not necessarily obey the Boltzmann thermal equilibrium, at least if the reaction time is shorter than the collision time. Instead, their internal degrees of freedom are populated based on the reaction paths rather than on the temperature of the surrounding environment. These out-of-LTE (*i.e.* non-LTE) populations encode information about the structural reaction dynamics and can manifest in the molecular spectra. The field of non-LTE spectroscopy has great potential to study these processes as the properties of the molecules producing the non-LTE spectroscopic signatures can shed the light on the dynamics of chemical reactions.^1–6^ The so-called transition state (TS) spectroscopy is a technique already widely used that employs the high-resolution non-LTE spectra of products to observe reaction processes that are hidden for the conventional spectroscopic methods.^7,8^ The novel high resolution non-LTE spectroscopic techniques allow decoupling of the vibrational and rotational degrees of freedom of molecules and thus control their vibrational and rotational populations, *e.g.* with rich vibrational and simplified rotational structures.^6,9–13^

The modern day study of non-LTE spectroscopy can be traced as far back as the 1930s to the original papers of Milne,^14^ and the many key papers from the decades following.^15–24^ The non-LTE spectroscopic effects play important role in high-resolution applications and there exist a number of accurate non-LTE spectroscopic and radiative transfer codes, see van der Tak *et al.*,^25^ Funke *et al.*,^26^ Pannier and Laux^27^ and references therein. As such, non-LTE spectra are often vital for the modelling of astrophysical problems, including planetary atmospheric properties,^28^ stellar atmospheres of solar system and exoplanets^29–36^ and the ISM.^25,37,38^

The non-local thermodynamic effects of the spectra of molecules has been of interest to chemists and astronomers alike for many years. A notable example of this is the 2011 work by Ferus *et al.*^2^ who studied the isomers of HCN within acetonitrile, formamide, and BrCN discharge. Using the features for both the HCN and HNC molecules from these spectra, Ferus *et al.*^2^ were able to calculate the ratios of molecules within the reactions and also the reaction path taken by the HNC molecule during the isomerization. In this work we explore this idea to study non-LTE spectral signatures of silylene (SiH_2_) produced from disilane (Si_2_H_6_).

Reaction properties of silylene, silane and disilane such as the rate constants for the formation, destruction and chemical pathways are important for plasma physics aspects such as silicon deposition.^39–41^ The ease of hydrogen transfer and high barriers in the saturated silicon system, leading to the ready formation of three-centre interactions and consequently the isomerisation reactions of Si_2_H_6_, are just as important to study as the elementary reactions.^42^ Silane containing reactions are also of importance for astrophysics, with the presence of SiH_4_ in IRC + 10216 discussed by Goldhaber and Betz,^43^ Kaiser and Osamura.^44^

**Fig. 1:**
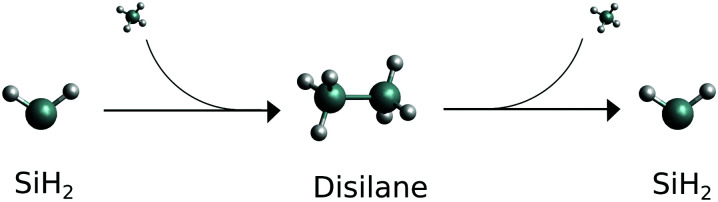
Schematic reaction path for the silylene. Molecular SiH_2_ is the starting material, fragment SiH_2_ is the product.

The complexity of the silane containing systems has been discussed elsewhere,^42,45^ with quantitative calculations proving particularly difficult. There were a number of *ab initio* studies of the structural properties of Si_2_H_6_*e.g.*,^46–53^ as well as of the formation and reactions involving this molecule.^54–58^ Agrawal *et al.*^53^ produced a global *ab initio* potential energy surface of disilane and used it to investigate the dissociation dynamics with classical trajectories. Márquez *et al.*^50^ reported a force field for Si_2_H_6_. The main isomer of Si_2_H_6_ has a staggered, ethane-like, structure (see Fig. 1) with a low barrier (1.2 kcal mol^−1^) to the eclipsed conformation.^59^ Si_2_H_6_ has been shown to have a local minimum as an inverted stable structure with one of the Si–H_3_ ‘umbrellas’ pointing to the center as well as a transition state with a similar inverted configuration.^46,60^ These structures are nominally asymmetric (*C*_s_ symmetry) but essentially acquiring the *C*_3v_ symmetry.

Thermal decomposition of Si_2_H_6_ has been extensively studied, both theoretically (mostly using RRKM, Rice–Ramsperger–Kassel–Marcus) and experimentally, with the reaction Si_2_H_6_ → SiH_2_ + SiH_4_ as the most common^53,56,57,61^ and important decomposition process of the excited disilane,^53,57,62–66^ and where Arrhenius parameters and rate constants have been reported (*e.g.* Bowrey and Purnell,^61^ Martin *et al.*,^63^ Mick *et al.*,^65^ Roenigk *et al.*^67^). However, it is also possible for the disilane molecule to dissociate homolytically, as was originally thought to be the main pathway owing to disilanes similarities with ethane, and form 
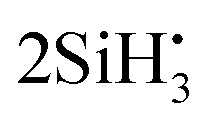
,^53,56,68^ or to undergo dehydrogenation to H_2_Si–SiH_2_, H_3_Si–SiH, or H_3_Si–H_2_.^53,54,57^ Disilane can even undergo double dehydrogenation to form Si_2_H_2_, however to our knowledge this has only been reported as the main product when undergoing photolysis at 193 nm.^69^ Yoshida *et al.*^57^ notes that the transition state for Si_2_H_6_ → SiH_2_ + SiH_4_ is 8.48 kcal mol^−1^ lower than the transition state for Si_2_H_6_ → H_3_SiSiH + H_2_, at 43.38 kcal mol^−1^ compared to 51.86 kcal mol^−1^.

The spectroscopy of SiH_2_ has been used to monitor the SiH_2_ + SiH_4_ → Si_2_H_6_ and Si_2_H_6_ → SiH_2_ + SiH_4_ reactions (see Fig. 1) and measure the corresponding rate constants and Arrhenius parameters by spectroscopically tracking electronic (*Ã*–*X̃*) transitions of SiH_2_.^39,46,65,70^ In these studies, the reconstructions of the amount of SiH_2_ relied on the assumption of the Boltzmann thermal distribution when estimating the population of the lower state. No account of the possible non-LTE population of SiH_2_ molecules after dissociation was made, which could potentially hamper the count of the SiH_2_ molecules and affect the reaction rates estimated. A similar experimental technique was used in Hertl and Jolly^71^ to monitor SiH_2_ in SiH_4_ plasma.

It is the second, dissociation, part of the reaction shown in Fig. 1 (Si_2_H_6_ → SiH_2_ + SiH_4_) we study in this work. More specifically, we show that (i) the (vibrational) populations of the molecules produced in reactions can be very different from the Boltzmann distribution and is important to take into account when interpreting spectroscopic measurements. That (ii) spectral shapes of the dissociated SiH_2_ can bear strong non-LTE character, very different from the LTE spectrum of an LTE SiH_2_ sample making it possible to distinguish between different reaction stages and even between different dissociation channels the silylene molecules it is produced from. In this work non-LTE spectra of SiH_2_ under conditions similar to dissociation processes expected in these experiments are modelled.

Recently we have computed an accurate ro-vibrational line list for SiH_2_, named CATS.^72^ It covers a large range of rotational and vibrational excitation, capable of modelling very hot spectra of this molecule (up to *T* = 2000 K) as part of the ExoMol database.^73^ The CATS line list was produced using the program TROVE,^74,75^ which solves the nuclear motion Schrödinger equation variationally. The ro-vibrational energies and corresponding wavefunctions were computed using an accurate, empirically refined potential energy surface (PES) of silane and a high-level *ab initio* dipole moment surface (DMS). The ro-vibrational probabilities (in the form of Einstein *A* coefficients) were computed using a high level *ab initio* dipole moment surface.

The study by Clark *et al.*^72^ forms the basis for the present work, where we utilize the CATS line list, wavefunctions, purpose-built numerical basis set, and the CATS computational TROVE setup to model non-LTE spectroscopic properties of SiH_2_ produced from dissociation of Si_2_H_6_ through different reaction channels. Using a simplified 1D Harmonic oscillator wavefunctions (see Pastorek *et al.*^6^) and more sophisticated 3D vibrational CATS wavefunctions from accurate variational calculations, the non-LTE ro-vibrational populations of SiH_2_ are generated and used to produce non-LTE spectroscopic spectra of different dissociation channels of disilane. To this end we investigate reaction topology connecting the global minimum of Si_2_H_6_ with the closest saddle points and local minima as well as the corresponding structural properties using a high level *ab initio* theory cc-pVTZ-F12/CCSD(T)-F12b^76,77^ employing the program MOLPRO2015.^78^

Theoretically, the non-LTE properties of dissociating molecules were studied by Band and Freed.^79^ In the present work we use general approach of Berry^80^ and Band and Freed,^79^ which assume no significant structural changes between the reactant and product nuclear configuration, along with the slow vibrational relaxation of the product^13^ to investigate non-LTE spectroscopic signatures of SiH_2_ produced from dissociation of Si_2_H_6_. In this paper we specifically consider situations where the vibrational relaxations are not achieved during the time of the experiment, so that the molecules still hold the memory of the structure during the reaction or dissociation. The rotation relaxation time however is much shorter and the rotational degrees of freedom can be usually assumed to satisfy the Bolzmann equilibrium.^13^

We also investigate possible non-LTE impact on the electronic *Ã*(0,2,0)–*X̃*(0,0,0) spectrum of SiH_2_. This is a favorite spectroscopic system for the detection of SiH_2_ due to the large Franck–Condon factor and the availability of suitable laser.^39,70,81–91^

The non-LTE absorption spectra of SiH_2_ are simulated using the (non-LTE) ExoCross program,^92^ where a new feature of non-Boltzmann populations was added. ExoCross has been previously used to model spectra of molecules in environments that can be characterized using two temperatures, vibrational and rotational.^93–96^

The paper is structured as follows. In Section 2 we describe the calculations of potential energy surfaces for the disilane and silylene structures. The theory used in this paper is described in Section 3. In Section 4.1 we calculate the 1D harmonic wavefunction population and use them to produce non-LTE spectra of SiH_2_ corresponding to different dissociation routes. In Section 4.2 we calculate the populations and subsequent non-LTE ro-vibrational spectra of SiH_2_ using the full 3D wavefunctions and describe the new TROVE methodology. A non-LTE electronic *Ã*(0,2,0)–*X̃*(0,0,0) spectrum of SiH_2_ is presented in Section 4.4. Conclusions are offered in Section 5.

## 2 Geometry optimisation and reaction topology of Si_2_H_6_

### 2.1 Disilane isomers

In order to better understand the reaction process of breaking Si_2_H_6_, the topology of Si_2_H_6_ has been investigated by performing a structural analysis of Si_2_H_6_ using a high level *ab initio* theory. This includes finding the global minimum (GM), local minima (LM), transition states (TS), reaction barriers as well as reaction paths, as detailed below. A reaction slice through the global PES of Si_2_H_6_ helps to indicate how likely local minima or transitions states were to be formed based on corresponding topology. These properties of Si_2_H_6_ were obtained using the geometry optimization and reaction path finder implemented in MOLPRO2015^78^ using the explicitly correlated coupled cluster method CCSD(T)-F12b^76,77^ with the F12-optimized correlation consistent basis set, VTZ-F12^97^ in the frozen core approximation. The calculations employed the diagonal fixed amplitude ansatz 3C(FIX)^98^ and a Slater geminal exponent value of *β* = 1.0*a*_0_^−1^.^99^ The auxiliary basis sets were chosen to be the resolution of the identity OptRI^100^ basis and the aug-cc-pV5Z/JKFIT^101^ and cc-awCV5Z/MP2FIT^102^ basis sets for density fitting. In the following this level of theory will be referenced to as VTZ/CCSD(T)-F12b.

We shall refer to different disilane isomers as dGM, dLM and dTS to distinguish them from the SiH_2_ fragments GM, LM and TS as discussed below.

The global minimum of SiH_2_ (dGM) has a symmetrical, staggered *D*_3d_ structure. The closest local minimum (dLM) has an inverted, *C*_3v_ structure. The lowest transition state, which will be referred to as dTS (TS1 in Becerra *et al.*^46^ and TS2 in Tonokura *et al.*^103^), has also inverted structure, just a few kJ mol^−1^ above dLM.^46^ These structures together with the corresponding optimized parameters for three geometries most relevant to our work are collected in Table 1. Our structural parameters compare well with that from the literature. The structure of the dGM has also been determined spectroscopically, with the equilibrium bond lengths *r*_Si–H_ = 1.492 Å, *r*_Si–Si_ = 2.331 Å and bond angles *β*_∠HSiSi_ = 110.3° and *α*_∠HSiSi_ = 108.6°.^104^ The structure of the deuterated isotopologue Si_2_H_5_D was reported as *r*_Si–H_ = 1.4874(17) Å, *r*_Si–Si_ = 2.3317(15) Å, *β*_∠HSiSi_ = 110.66(16)°.^105^

**Table 1:** An overview of the Si_2_H_6_ molecules discussed in the paper. The subscript L denotes atoms to the LHS of the Si–Si bond in the figures shown, and the subscript R denotes atoms to the RHS

Isomer	LM	TS	GM	Becerra *et al.*^46^		
Molecule	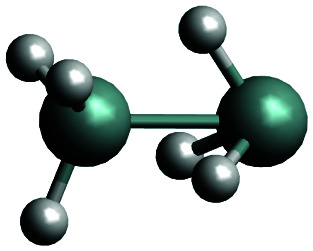	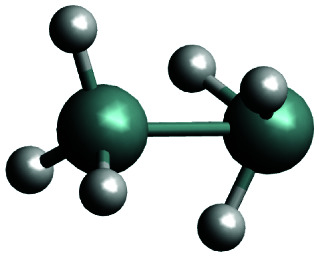	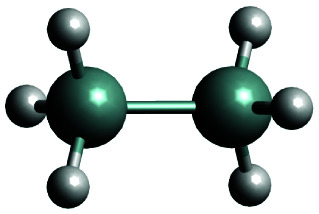	LM1	TS1	GM
Si–Si (Å)	2.467	2.489	2.341	2.470	2.485	2.346
Si_R_–H_R_ (Å)	1.532	1.499	1.482	1.525	1.493	1.479
Si_L_–H_L_ (Å)	1.480	1.480	1.482	1.478	1.478	1.479
∠H_R_SiSi (°)	55.4	64.9	110.2	—	—	110.24
∠H_L_SiSi (°)	110.7	110.0	110.2	110.7	110.2	110.24
∠H_R_SiH_R_ (°)	90.9	100.6	108.6	91.4	102.6	—
∠H_L_SiH_L_ (°)	108.3	109.7	108.6	108.2	108.8	—
*ω*_b_ (cm^−1^)	940	940	940			
*ω*_s(L)_ (cm^−1^)	2241.69	2245.36	2240.10			
*ω*_s(R)_ (cm^−1^)	2039.12	2170.66	2240.10			

**Fig. 2:**
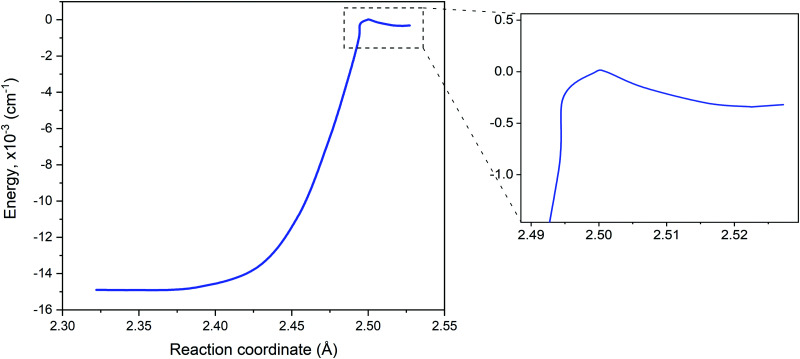
Potential energy curve for the reaction path from disilane global minimum (dGM), left, through the transition state (dTS) onto the local minimum (dLM), right. A zoom of dLM to dTS path is inset. The dLM is 344.22 cm^−1^ lower than the energy of the dTS.

The reaction path connecting the disilane isomers dGM, dLM and dTS is shown in Fig. 2. A zoom of the dLM side is shown as inset. The energies of the global and local minima are 178 kJ mol^−1^ and 4.1 kJ mol^−1^ below the transition state, respectively. The energy and geometry information for dLM, dGM, dTS are collected in Table 2. The results calculated compare well with both the results of Becerra *et al.*^46^ and Sakai and Nakamura,^60^ albeit both vary for the dGM structure by 36 kJ mol^−1^.

**Table 2:** The total and relative energies for the three disilane structure. Literature values from Becerra *et al.*^c^^ 46^ and Sakai and Nakamura^60^

	Energy, *E*_h_	Rel. energy,^d^ kJ mol^−1^
	**dGM**	
Calculated	−581.728134	−178.00
Becerra	−236.0	−214.50
Saki	−581.53962	−214.68715
	**dTS**	
Calculated	−581.660338	0.00
Becerra (TS1)^a^	−48.5	0.00
Saki (TS2)^a^	−581.45785	0.00
	**dLM**	
Calculated	−581.661906	−4.12
Becerra (LM1)^b^	−51.3	−3.00
Saki (compl. 2)^b^	−581.45837	−1.3652601

a TS2 in Sakai and Nakamura^60^ is the same as TS1 in Becerra *et al.*^46^

b Complex 2 in Sakai and Nakamura^60^ is the same as LM1 in Becerra *et al.*^46^

c Relative to SiH_2_ + SiH_4_, kJ mol^−1^.

d Relative to dTS.

The dLM isomer of Si_2_H_6_ has a shallow potential with a very low barrier to dTS of 344 cm^−1^. It can be also recovered using lower levels of theory, for example, using MP2/6-311G(d,p)^46^ and even with the UFF force fields implemented in Avogadro 1.2.0^106^*via* the steepest descent method and 4 steps per update.

### 2.2 The silylene fragments

With the aim to give more quantitative information on the structural and dynamical properties of five SiH_2_ fragments from Si_2_H_6_, the fragments are described as follows. GM is an SiH_2_ fragment from the global minimum structure (dGM); LM-L is an SiH_2_ fragment from the left hand side (LHS) of the local minimum structure (dLM) with the Si–H_3_ umbrella group pointing outside; LM-R is an SiH_2_ fragment from the right hand side (RHS) of the dLM structure with Si–H_3_ pointing inside; TS-L is an SiH_2_ fragment from the LHS of the transition state structure (dTS), Si–H_3_ umbrella group points outside; and TS-R is an SiH_2_ fragment from the RHS of the dTS structure, Si–H_3_ umbrella group pointing inside. The structural parameters and structures are shown in Table 3 and the energies are shown in Table 2. The harmonic frequencies for the disilane molecules were computed with MOLPRO using the TVZ/CCSD(T)-F12b level of theory. The results from the frequency analysis are shown in Table 3 along with a comparison with literature (both experimental, if available, or theoretical data is shown). The symmetry of dGM is *D*_3d_ whereas the symmetry for the dLM and dTS are both *C*_3v_. The columns titled “Theory” were calculated in this work, and the degenerate states have been removed, with an average calculated if there were any differences owing to computational errors associated with the lower symmetry used by MOLPRO. All frequencies of dLM are positive thus confirming that it is a minimum with a stable structure. The ‘negative’ (or imaginary) harmonic frequency of dTS is −551.6 cm^−1^.

**Table 3:** The calculated and available literature vibrational frequencies in cm^−1^ for the three Si_2_H_6_ molecules

dGM, *D*_3d_				dTS, *C*_3v_				dLM, *C*_3v_	
Calculated		Literature^46^		Calculated		Literature^46,67^		Calculated	
Mode	Freq.	Mode	Freq.	Mode	Freq.	Mode	Freq.	Mode	Freq.
*E*_u_	2247.78	*E*_u_	2179	*E*	2252.06	*A*	2181	*E*	2244.30
*A*_1g_	2241.03	*A*_1g_	2163^a^	*A*	2238.66	*E*	2169	*A*	2239.08
*E*_g_	2239.28	*E*_u_	2155	*E*	2176.47	*A*	2105	*A*	2050.89
*A*_1u_	2232.30	*A*_1u_	2154	*A*	2161.85	*A*	2087	*E*	2027.35
*E*_u_	962.35	*E*_g_	941^a^	*E*	995.37	*A*	1585	*E*	1089.15
*E*_g_	948.37	*E*_u_	940	*E*	966.95	*A*	960	*E*	978.33
*A*_1g_	931.91	*A*_1g_	920^a^	*A*	902.23	*A*	949	*A*	956.33
*A*_1u_	859.02	*A*_1u_	844	*E*	570.16	*A*	927	*A*	667.30
*E*_g_	636.87	*E*_g_	628^a^	*A*	353.88	*A* & *E*^b^	925	*E*	618.92
*A*_1g_	438.76	*A*_1g_	432^a^	*E*	319.45	*E*	145	*A*	432.13
*E*_u_	372.85	*E*_u_	379	*A*	316.43	*A*	128	*A*	389.31
*A*_1u_	136.56	*A*_1u_	128	*A*	−551.59	*E*	93	*E*	277.91

a Confirmed by the experimental gas phase Raman spectrum of Durig and Church.^59^

b Although there are three lines of 925 cm^−1^, there are no triply degenerate states in the *C*_3v_ symmetry. Roenigk *et al.*^67^ assign one 925 cm^−1^ to *ν*_15_, which in the dGM symmetry is an *E* state degenerate with *ν*_16_. The dTS *ν*_16_ can be found at 960 cm^−1^. The other two 925 cm^−1^ line were assigned to *ν*_9_ and *ν*_10_ (*E*).

## 3 Modelling the non-LTE populations of SiH_2_

We now consider the decomposition reaction Si_2_H_6_ → SiH_2_ + SiH_4_ shown in Fig. 1 and model the vibrational populations of the product SiH_2_ assuming that the corresponding relaxation time to LTE is much longer than the time of the spectroscopic experiment. We aim at simulating non-LTE IR spectra of SiH_2_ using these populations to demonstrate their impact on the spectral shape of dissociated species.

In our description of the non-LTE vibrational population of the dissociated molecule we follow the polyatomic Franck–Condon type approximation by Band and Freed^79^ and Berry^80^ based on the structural differences between reactant and product assuming no significant change in nuclear configuration of the molecule. In order to connect the product (gas phase SiH_2_) to an initial structure of Si_2_H_6_ through the dissociation process, we assume that the dissociation happens instantaneously, *i.e.* the initial configuration of the product SiH_2_ corresponds to the structural parameters (bond lengths Si–H and inter-bond angles ∠HSiH) of SiH_2_ as a fragment of Si_2_H_6_, for which the parameters collected in Table 1 are used. For example, for the dissociation from the dGM structure, the initial configuration of the gas phase SiH_2_ is assumed to be *r*_Si–H_ = 1.482 Å, *α*_∠HSiH_ = 108.6°. Naturally, this is a very deformed geometry comparing to the equilibrium structure of the gas phase SiH_2_, *r*_e_ = 1.5137 ± 0.003 Å and *α*_e_ = 92.04 ± 0.05°.^107^ After being dissociated, in relaxing to be a free molecule, the fragment SiH_2_ has added vibrational energy and is hence in a situation when it's vibrational populations do not match the LTE distribution for corresponding temperature of the surroundings, at least for the vibrational degrees of freedom.

Experience shows that the rotational equillibration time is usually very short and we can thus safely assume the LTE conditions for the rotational degrees of freedom with the rotational temperature the same as the temperature of the surroundings (see also *e.g.* Dudás *et al.*^13^). The vibrational population however is not in the thermal equilibrium and therefore no sensible vibrational temperature could be associated with the corresponding population.

The non-LTE population *N*_*J*,*k*,*ν*_(*T*) of a ro-vibrational state |*J*, *k*, *ν*〉 is then given by:^6^1
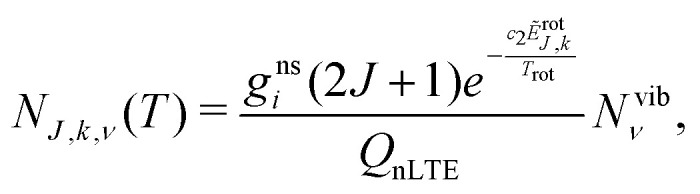
where *N*^vib^_*ν*_ is a non-LTE vibrational population, *T*_rot_ is the rotational temperature, *J* is the total angular momentum quantum number; *k* is a generic rotational quantum number, *e.g.* the projection of the total angular momentum on the molecular *z* axis; is a generic vibrational quantum number/label, *e.g.* a combination (*ν*_1_,*ν*_2_,*ν*_3_) to describe vibrational states of a triatomic molecule; *g*^ns^_*i*_ is the nuclear spin degeneracy; *T* is the temperature, *c*_2_ is the second radiation constant. In eqn (1), *Ẽ*^rot^_*J*,*k*_ is the rotational part of the ro-vibrational energy term value approximated as

2 *Ẽ*_*J*,*k*,*ν*_ = *Ẽ*^vib^_*ν*_ + *Ẽ*^rot^_*J*,*k*_,

where *hc*·*Ẽ*^vib^_*ν*_ is the vibrational (*J* = 0) energy (‘band center’) and *hc*·*Ẽ*_*J*,*k*,*ν*_ is the total ro-vibrational energy. The non-LTE partition function *Q*_nLTE_ in eqn (1) is given by3
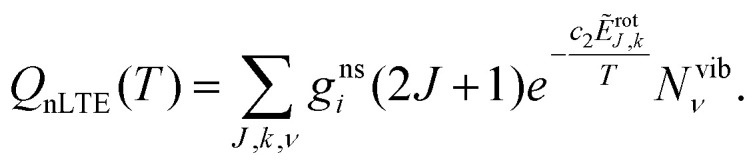
Our aim is to calculate the vibrational populations *N*_*v*_ ≡ *N*^vib^_*ν*_ of SiH_2_ as produced by instantaneous (vertical) dissociation from three structures, dGM, dLM and dTS. In case of dLM and dTS, the SiH_2_ fragment can originate from any of the two different sides of their inverted structures, which should be taken into account. We therefore have to consider five different fragments, as shown in Table 4.

**Table 4:** Five SiH_2_ fragments considered for the non-LTE analysis

SiH_2_ fragment name	Formation & structure
GM	From dGM, two equivalent SiH_3_
TS-R	From dTS, SiH_3_ pointing inside
TS-L	From dTS, SiH_3_ pointing outside
LM-R	From dLM, SiH_3_ pointing inside
LM-L	From dLM, SiH_3_ pointing outside

Let us assume that the Si_2_H_6_ is LTE and hence is in its ground vibrational state at the moment of dissociation, while the fragment SiH can end up in any vibrationally excited state ≡ (*ν*_1_,*ν*_3_) with some transition probability giving rise to the vibrational population *N*^vib^_*ν*_. On top of that we also assume a full separation of the stretching Si–H and bending ∠HSiH modes inside Si_2_H_6_ in its ground vibrational state. Possible consequences of deviation from these approximations are discussed below.

Under the assumptions made we define the vibrational population of the SiH_2_ fragment as a Franck–Condon factor for a vertical transition from the ground vibrational state of disilane Si_2_H_6_ to SiH_2_ + SiH_4_ with the gas phase (g.ph.) SiH_2_ transferred to some vibrational state |(g.ph.)〉 = |*ν*_1_,*ν*_2_,*ν*_3_〉. In the approximation of the full separation of the fragment SiH_2_ from the rest of Si_2_H_6_, the population of SiH_2_ can be represented as an overlap between the ground state wavefunction |*ν*′′ = 0(fragment)〉 of a fragment SiH_2_ and that of the corresponding vibrational state |*ν*′ = (g.ph.)〉 of the gas phase SiH_2_ as given by

4 *N* = |〈0(fragment)|*ν*(g.ph.)〉|^2^.

Here the spacial wavefunction of the SiH_2_ fragment (*i.e.* a combination of two adjacent Si–H bonds in disilane with an angle between them forming the dissociating SiH_2_) is projected on vibrational eigenfunctions of the gas phase SiH_2_ to give the corresponding populations of SiH_2_. The highest populated energy level will have the largest overlap between these wavefunctions.

The calculated temperature dependent populations *N*_*J*,*k*,*ν*_(*T*) in eqn (1) can be then combined with a molecular line list for SiH_2_ to simulate absorption or emission spectra of this molecule under the non-LTE conditions as defined by *N*_*J*,*k*,*ν*_(*T*) in eqn (1). Here we use the ExoMol line list CATS by Clark *et al.*^72^ as provided by ExoMol (www.exomol.com). Technically this is done by incorporating the non-LTE vibrational densities *N* into the ExoMol States file as described in Section 4 (the ExoMol file formats are discussed extensively elsewhere^73^). A non-LTE spectrum of SiH_2_ for given *T* and *P* is then calculated using CATS' Einstein *A* coefficients with the ExoCross program,^92^ where a new non-LTE option has been implemented as part of this work. The rotational populations are assumed to be in LTE according with eqn (1).

## 4 Computing vibrational populations of SiH_2_

Two approaches were used for the calculation of the population densities of the fragment SiH_2_. One approach – named the decoupled 1D approach – is where the 3D wavefunctions of the fragment as well as of free SiH_2_ are represented by products of 1D parts with the harmonic oscillators as wavefunctions. This simplified model is mainly used to illustrate the idea of our non-LTE treatment. The second, more accurate approach – named the 3D approach – is based on the full 3D vibrational wavefunctions computed using the variational program TROVE.^74^ Both approaches are presented in the following in order to assess and compare the accuracy achieved.

### 4.1 The 1D approach

A vibrational state |*ν*〉 = |*ν*_1_,*ν*_2_,*ν*_3_〉 of SiH_2_ is characterized by the three (normal mode) quantum numbers *ν*_1_, *ν*_2_ and *ν*_3_ corresponding to the two stretching modes (*ν*_1_ and *ν*_3_) and one bending mode (*ν*_2_) of the SiH_2_ molecule. The 1D approach considers the stretching Si–H_1_, Si–H_2_ and bending ∠HSiH modes, both of the molecular fragment and gas phase SiH_2_ molecules, as fully independent and described by one dimensional (1D) wavefunctions under the harmonic approximation, as given by:5
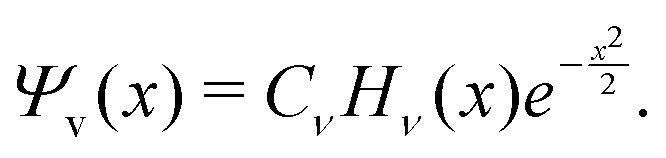
Here *x* is a dimensionless coordinate describing either the stretching *r* = *r*_Si–H_ or bending *α* = *α*_∠H–Si–H_ coordinate as follows:6
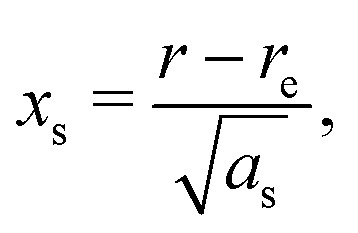
7
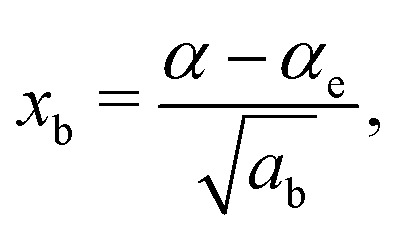
with8
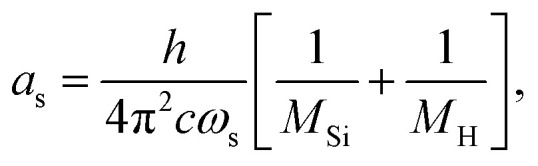
9
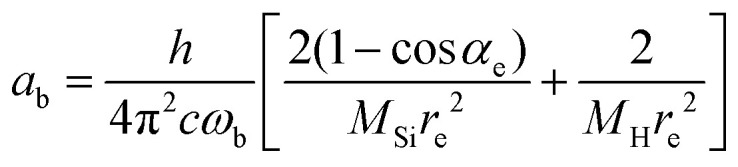
and *ω*_s_ = *ω*_Si–H_, *ω*_b_ = *ω*_∠HSiH_. In eqn (5)*H*(*x*) is a Hermite polynomial and *C*_*v*_ is the corresponding normalization constant.

The constants *a*_s_ and *a*_b_ correspond to inverse masses of the vibrational part of a free three-atomic molecule expressed in terms of the internal coordinates *r*_1_, *r*_2_, *α* (see, *e.g.* Sutcliffe and Tennyson,^108^ Yurchenko *et al.*^109^).

A 1D population for the corresponding mode of the gas phase SiH_2_ molecule is given by eqn (4) with |*ν*〉 = *Ψ*_*v*_(*x*). The different disilane fragments have different structural parameters *r*_e_, *α*_e_, *ω*_s_ and *ω*_b_, see Tables 1 and 3, and thus lead to different ground state vibrational 1D wavefunctions |0(fragment)〉 (stretching or bending) and hence result in different vibrational populations *N*_*ν*_ of the gas phase SiH_2_ according with eqn (4).

For each of the three modes (two stretching and one bending), 1D wavefunctions of the gas phase SiH_2_ for 30 vibrational states from *ν*_0_ up to *ν*_29_ were calculated. These 1D wavefunctions were then numerically integrated with the corresponding ground state 1D wavefunctions |*ν* = 0〉 of the fragment in question.

The total vibrational population *N*_*ν*_1_,*ν*_2_,*ν*_3__ in this approximation is then given by a product10
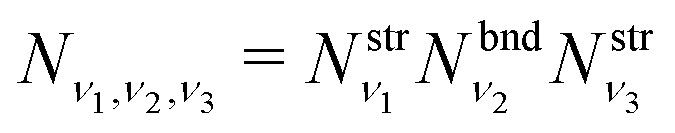


where 
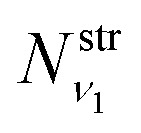
 and 
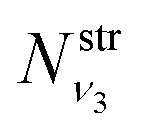
 are obtained using the stretching harmonic oscillators wavefunctions |*ν*_1_〉 and |*ν*_3_〉, while 
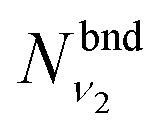
 is obtained using the corresponding bending harmonic oscillator wavefunction |*ν*_2_〉. The independent treatment of the two stretching populations is partly justified by the local mode character of the vibrational degrees of freedom of SiH_2_ due to the near 90° bond angle (see, *e.g.* Jensen^110^ and Clark *et al.*^72^). The asymmetric vibrational modes of SiH_2_ (*B*_2_ in *C*_2v_) are non populated in this 1D approximation. This is because for the parallel nature of the Franck–Condon transitions from the ground vibrational state of disilane, which is fully symmetric (*A*_1_), only symmetric states of SiH_2_ give rise to non-zero integrals in eqn (4). For example, the vibrational population of the (2,1,0) state (2 stretching and 1 bending quanta of *A*_1_) of the GM fragment is obtained as a product of 
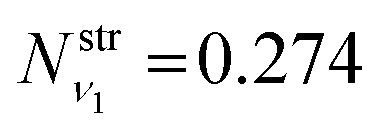
, 
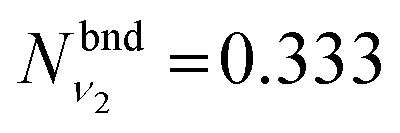
 and 
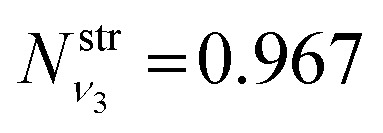
, respectively, resulting in *N*_(*ν*2,*ν*1,*ν*0)_ = 0.088, while the population of the *B*_2_-type (0,0,1) vibratitonal state is assumed to be zero. The populations *N*_*ν*1,*ν*2,*ν*3_ are pre-calculated for each vibrational state (*ν*_1_,*ν*_2_,*ν*_3_) of SiH_2_ and added to the CATS State file to be used in non-LTE simulations (see below, Section 4.3). All the vibrational populations computed and used as part of this work are provided in the ESI.†

#### 4.1.1 1D populations and spectra

**Fig. 3:**
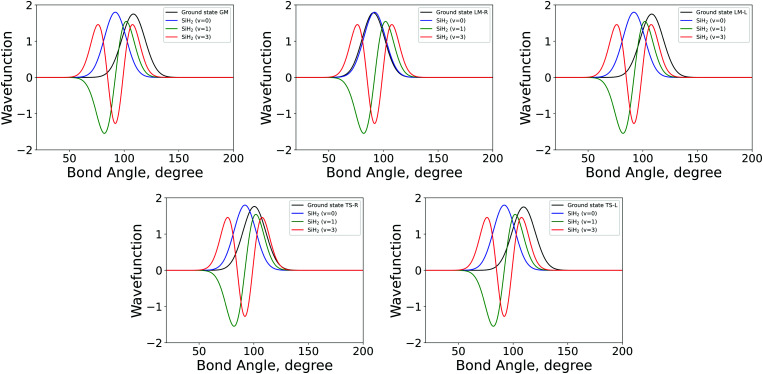
The 1D bending mode harmonic wavefunctions for the five SiH_2_ fragments described in Table 1 as a function of bond angle in degrees. Ground state fragment (black) compared with the LTE |*ν* = 0〉 (blue), |*ν* = 1〉 (green), |*ν* = 2〉 (red).

**Fig. 4:**
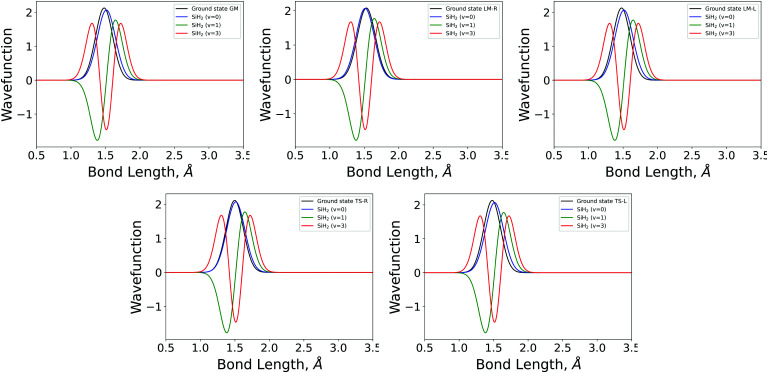
The 1D stretching mode harmonic wavefunctions for the five SiH_2_ fragments described in Table 1 as a function of bond length in Angstrom. Ground state fragment (black) compared with the LTE |*ν* = 0〉 (blue), |*ν* = 1〉 (green), |*ν* = 2〉 (red).

**Fig. 5:**
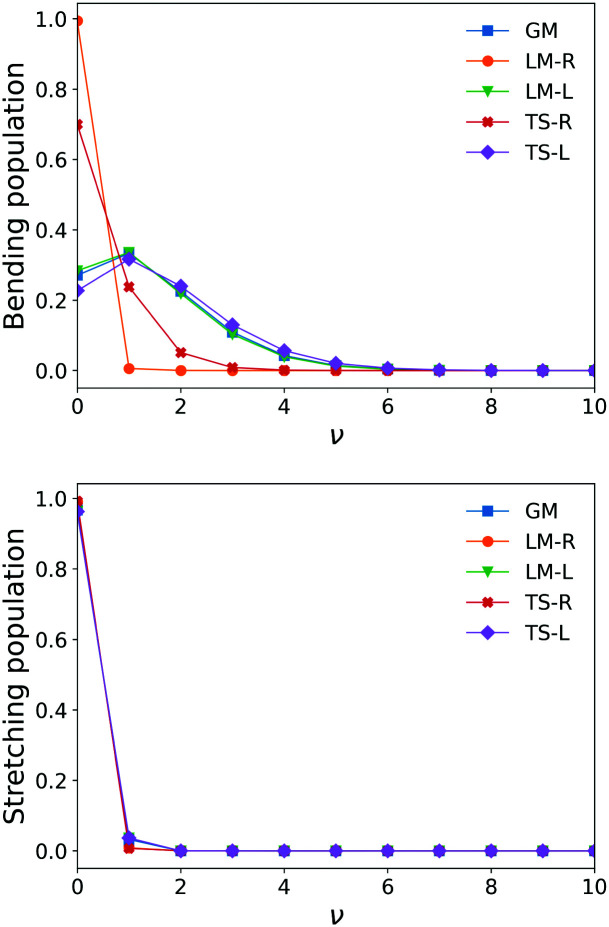
Stretching and bending mode 1D populations *N*_v_ for the five SiH_2_ fragments from *ν* = 0–*ν* = 10.

Examples of overlapping bending mode Harmonic wavefunctions used in calculations of populations for *ν* = 0, 1 and 2 of the five fragments are shown in Fig. 3 and 4 for the bending and stretching modes respectively. In all cases the black curve represents the ground state wavefunction for the non-LTE fragment, and the blue, green and red line show the *ν* = 0, *ν* = 1 and *ν* = 2 LTE wavefunctions of gas phase SiH_2_.^72^ The corresponding 1D populations as an integral of the overlaps between the LTE and non-LTE wavefunctions are plotted in Fig. 5 for the bending and stretching modes of the five fragments. The stretching populations exhibit a typical Boltzmann-like distribution with the ground vibrational state *ν* = 0 as the mostly populated in all five cases. This is expected because their equilibrium bond lengths are rather similar to that of the gas phase SiH_2_. In case of the bending populations, only LM-R and TS-R have *ν* = 0 to be with the highest populations, while for LM-L, TS-L and GM the *N* distributions exhibit strong non-LTE character with *ν* = 2 to be almost as populated as the ground vibrational state *ν* = 0.

The shapes and positions of the curves in Fig. 3 and 4 match with the parameters from Table 1. The larger *ω* used for the bending modes manifests itself as wider curves, while the black curves are all centred around the equilibrium bond angles and length listed in Table 1.

The similarity between the populations of the LM-L, TS-L and GM fragments is expected owing to their similar structural parameters. It is interesting to see the most populated vibrational levels of LM-R and TS-R are always lower than the vibrational levels of LM-L, TS-L and GM.

**Fig. 6:**
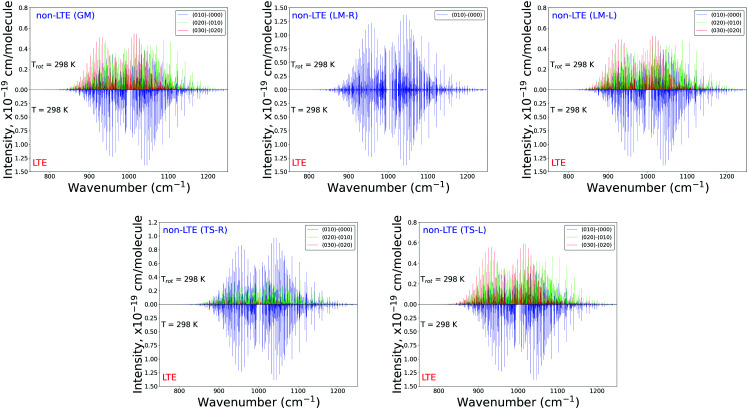
Non-LTE spectra of SiH_2_ at *T*_rot_ = 296 K in the 1000 cm^−1^ (10 μm) region corresponding to five vibrational populations GM, TS-L, TS-R, LM-L and LM-R (upper displays of each figure) and compared to the same spectra simulated using the LTE population at *T* = 296 K. The non-LTE populations were obtained using the 1D approach (see text). Only the strongest bands are shown. The CATS line list was used.

**Fig. 7:**
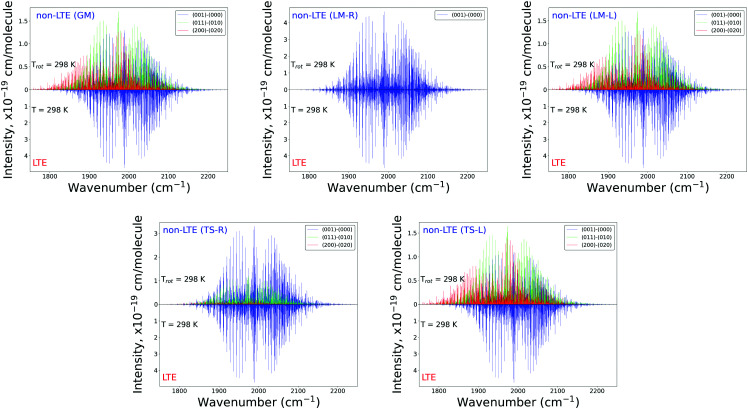
Non-LTE spectra of SiH_2_ at *T*_rot_ = 296 K in the 2000 cm^−1^ (5 μm) region corresponding to five vibrational populations GM, TS-L, TS-R, LM-L and LM-R (upper displays of each figure) and compared to the same spectra simulated using the LTE population at *T* = 296 K. The non-LTE populations were obtained using the 1D approach (see text). Only the strongest bands are shown. The CATS line list was used.

Fig. 6 and 7 show the 1000 cm^−1^ and 2000 cm^−1^ (10 μm and 5 μm) bands for the SiH_2_ absorption spectrum, respectively, simulated using the non-LTE densities from Fig. 5 for all five cases considered and compared to the LTE scenario assuming the (rotational) temperature of *T* = 296 K and using the CATS line list. The strongest bands are indicated using different colours. Fig. 6 focuses on the 1000 cm^−1^ band. Most of the non-LTE spectra contain bending hot bands (020)–(010) and (020)–(010), which are stronger than the fundamental band (000)–(000). It can be seen that the *P* and *R* branches of the non-LTE spectra are shifted to lower wavenumbers in the GM, LM-L and TS-L spectra. In the TS-R and LM-R spectra the bands are not shifted, with the LM-R spectrum having only the fundamental (010)–(000) band visible.

The plots in Fig. 7 show the 2000 cm^−1^ band in the region of the polyad (100)/(020)/(001) for the five fragments, with the strongest fundamental band (001)–(000). The non-LTE intensities of the hot bands (011)–(010), (200)–(020) are found to be comparable to the intensities of the (001)–(000) band. The *Q* branch is clearly shifted for the GM, TS-L and LM-L molecules. The band is less shifted for the TS-R and LM-R fragments, but owing to the increased similarity between the fragment and molecular structures with TS-R and LM-R this is to be expected. Only the main polyad system (100)/(020)/(001) is visible for the TS-R spectrum (indicated as (001)–(000) in Fig. 7).

With the equilibrium structures of the TS-R and LM-R fragments being similar to the equilibrium structure of SiH_2_, their non-LTE spectra are expected to be a similar spectrum to LTE. Indeed, for the 1D harmonic approach their *P*, *Q* and *R* branches maintain the expected LTE intensities for both the 1000 cm^−1^ and 2000 cm^−1^ bands.

### 4.2 The 3D approach for vibrational populations using an accurate variational method

In a full 3D approach, the ground state wavefunction |0,0,0(fragment)〉 represents an SiH_2_ fragment of an 18D ground state vibrational wavefunction of Si_2_H_6_:*ϕ*_Si2H6_ = |0,0,0(SiH_2_)〉 |0,0,0,0,0,0,0,0,0,0,0,0,0,0,0(SiH_4_)〉.Here we assume the approximation that the corresponding three modes (Si–H_1_, Si–H_2_ and ∠H_1_SiH_2_) are independent from the rest of the molecule so that all other modes, not relevant for the gas phase SiH_2_, can be eliminated (integrated out), including the reaction coordinate and vibrational modes of SiH_4_. This is in line with the assumptions used previously by Band and Freed^79^ and Berry.^80^ Apart from this approximation we will treat the SiH_2_ fragment as accurate as possible. The corresponding wavefunction |0,0,0(fragment)〉 is obtained by solving a 3D vibrational Schrödinger equation for these three degrees of freedom with a realistic PES obtained using a high level of *ab initio* theory (the same as above, VTZ/CCSD(T)-F12b with MOLPRO).

The vibrational populations are then modelled using the Franck–Condon integrals as follows:

11 *N*_*ν*1,*ν*2,*ν*3_ = |〈0,0,0(fragment)|*ν*_1_, *ν*_2_, *ν*_3_(g.ph.)〉|^2^,

with the sum of all populations over all states totalling 1. In this equation, |*ν*_1_,*ν*_2_,*ν*_3_(g.ph.)〉 is an accurate vibrational wavefunction of a gas phase SiH_2_ molecule, obtained by solving the vibrational Schrödinger equation with an accurate PES. We use the TROVE variational program and the refined PES of SiH_2_ by Clark *et al.*^72^ to generate |*ν*_1_,*ν*_2_,*ν*_3_(g.ph.)〉 for all vibrational excitations required. For the details on the TROVE calculations see below and also Clark *et al.*^72^

In order to simplify the 3D integration in eqn (11), the variational wavefunction |0,0,0(fragment)〉 is obtained using the same vibrational basis set as the variational solution of the gas phase SiH_2_. By taking advantage of the compatibility of the orthogonality of the basis sets, the Franck–Condon factors are then given by as a sum of products12
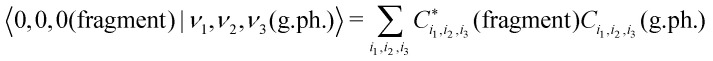
of the corresponding eigen-coefficients *C*_*i*1,*i*2,*i*3_(fragment) and *C*_*i*1,*i*2,*i*3_(g.ph.), obtained variationally in independent calculations using a new implementation in TROVE.

TROVE uses optimized non-standard vibrational basis sets, generated numerically by solving 1D Schrödinger equations for realistic 1D potentials.^74^ This procedure allows producing compact basis functions optimized for a specific problem. In our case, the PESs of the corresponding five fragments and of the gas phase SiH_2_ are different and therefore the generated basis sets would be different and even not orthogonal. We therefore implemented a feature in TROVE allowing to read and use externally generated basis functions. Of course all relevant calculation setups must be compatible, including the numerical grids used for the stretching and bending modes and their sizes. Using foreign basis sets certainly degrades their quality. However, since we are only interested in fragments' ground state wavefunctions, this degradation can be mitigated by including enough basis functions. Our typical 1D basis sets contain 12–24 functions (see details below), which should be more than enough to obtain a converged ground state solution even with non-optimized basis sets.

#### 4.2.1 PESs of SiH_2_ fragments

For our new 3D populations corresponding to dissociations from the fragments, five PESs were generated as follows. We assume that PESs of a dissociating Si_2_H_6_ molecule can be approximated as a sum of two independent fragments:*V*_Si2H6_ = *V*_SiH2_(*r*_1_, *r*_2_, *α*) + *V*_rest_,where the individual stretching and bending modes of SiH_2_ fragments are fully separable:

13 *V*_SiH2_(*r*_1_, *r*_2_, *α*) = *f*_str_(*r*_1_) + *f*_str_(*r*_2_) + *f*_bnd_(*α*).

The stretching part of the potential is given by a Morse-like expansion14
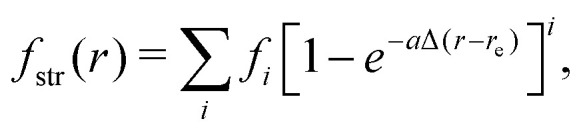
while the bending part is a Taylor-type expansion in terms of the displacement from the corresponding equilibrium value:15
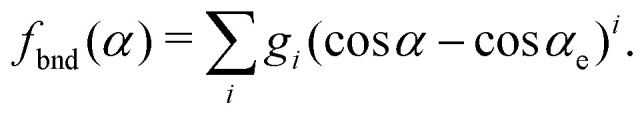
The expansion constants *f*_*i*_ and *g*_*i*_ representing fragments' potential energies *V*_SiH2_(*r*_1_,*r*_2_,*α*) were obtained by fitting eqn (14) and (15) to the *ab initio* data computed as 1D slices on the global surfaces for the five fragments from Si_2_H_6_ (dGM, dLM and dTS) using VTZ/CCSD(T)-F12b consisting of 24 bending and 34 stretching geometries, distributed around the corresponding equilibria. They are listed in Table 5.

**Table 5:** The *ab initio* potential parameters defining the 1D potential of different structures used in TROVE calculations. The units are cm^−1^, Å and radians, unless specified

Parameter	GM	TS-L	TS-R	LM-L	LM- R
*r*_e_, Å	1.48218	1.47971	1.49888	1.47982	1.51611
*α*_e_, deg	108.701	108.938	103.268	108.264	92.287
*a*, Å^−1^	1.29065	1.29110	1.22364	1.27911	1.27050
*g*_1_	0	0	0	0	−8132.4642
*g*_2_	80964.377	90039.635	101613.84	93608.413	168296.56
*g*_3_	−117331.83	−126539.98	−159054.10	−129420.43	−299843.15
*g*_4_	139212.98	168090.27	182459.45	305247.60	324286.87
*f*_1_	0	0	0	0	−793.07685
*f*_2_	43617.985	43796.459	45462.423	22217.055	19382.230
*f*_3_	−6594.4391	−6937.7586	−8917.729	−3824.8354	−2298.2220
*f*_4_	2809.6655	2714.6696	3750.0966	1407.7267	2233.5205

**Fig. 8:**
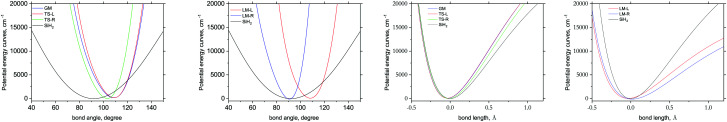
1D potential energy cuts representing different stretching Si–H (*x*-axis = bond length) and bending (*x*-axis = bond angle) H–Si–H modes of different fragments of three Si_2_H_6_ isomers, dGM, dLM and dTS, compared to the corresponding cuts of the gas phase SiH_2_ species.

Fig. 8 illustrates the *ab initio* PESs of different fragments as 1D cuts for the stretching and bending modes compared to the corresponding cuts of the gas phase SiH_2_ molecule. The bending cuts have especially different shapes with shifts to larger equilibrium angles and much steeper PESs. The differences in the stretching cuts are less pronounced. These features are important for the non-LTE behaviour of the corresponding excited states populations, with the bending degree of freedom to have stronger non-LTE character than stretching.

#### 4.2.2 Vibrational calculations

The vibrational wavefunctions of SiH_2_ were computed using the variational nuclear motion program TROVE with the same setup as in Clark *et al.*^72^ Details of the TROVE methodology are discussed extensively elsewhere.^74,111–113^ Here, we give a brief outline of the main calculation steps. The TROVE kinetic energy operator is Taylor expanded up to sixth order around the SiH_2_ equilibrium geometry in terms of linearized coordinates.^114^ The primitive basis set is constructed from 1D mode numerical basis functions using the Numerov–Cooley approach^115,116^ by solving three 1D Schrödinger equations, for each vibrational degree of freedom. The stretching basis functions are then improved by solving a 2D Schrödinger equation for a reduced stretching Hamiltonian. The resulting stretching eigenfunctions are contracted, classified according with the *C*_2v_(*M*) symmetry group^114^ using an optimized symmetrization procedure^112^ and combined with the bending primitive basis functions to form our final, symmetry-adapted 3D vibrational basis set. The basis set coverage is defined by the polyad number cut-off

16 *P*= 2(*ν*_1_ + *ν*_3_) + *ν*_2_ ≤ 24,

where *ν*_1_ and *ν*_3_ are the stretching and *ν*_2_ is the bending quantum numbers, with the maximal excitations 12, 12 and 24 respectively.

For the gas phase SiH_2_ calculations we employed the empirically refined PES by Clark *et al.*^72^ For the five SiH_2_ fragments only the vibrational ground state wavefunctions |0,0,0〉 were computed using the same setup and utilizing the basis functions from the g.ph. calculations as described above, but for the fragments' *ab initio* PESs from eqn (13).

#### 4.2.3 Vibrational populations of SiH_2_

The 3D vibrational populations *N*_*ν*_1_,*ν*_2_,*ν*_3__ in eqn (11) were computed using the corresponding TROVE eigenfunctions *via*eqn (12).

**Fig. 9:**
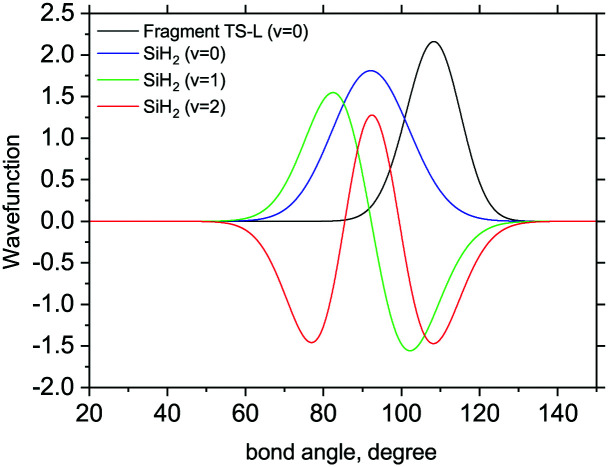
3D Bending primitive wavefunctions of SiH_2_ compared to the ground state 3D bending wavefunctions for Fragment TS-L. Calculated with TROVE.

**Fig. 10:**
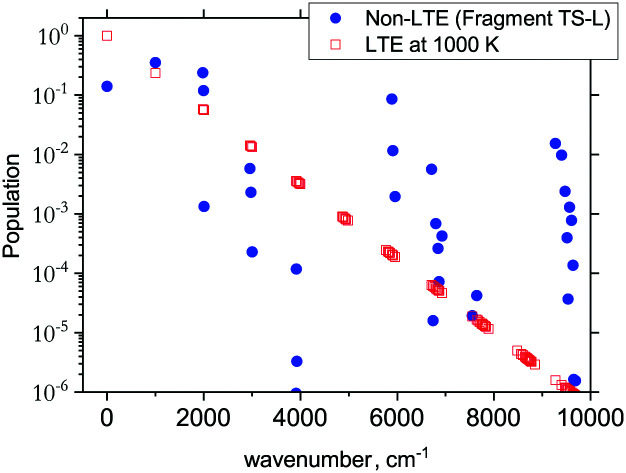
The 3D non-LTE vibrational populations of SiH_2_ for the dissociation from TS-L (blue circles) as a function of the vibrational term values compared to Boltzmann distribution of SiH_2_ vibrational states at *T* = 1000 K.

The 3D results generally agree with the 1D case. As an example, Fig. 9 shows the TS-L case: the bending primitive wavefunctions of the gas phase SiH_2_ are compared to the *ν* = 0 bending wavefunction generated for the PES of TS-L. Fig. 10 shows the corresponding non-LTE vibrational populations as a function of the corresponding energies for the same TS-L scenario of the 3D vibrational populations. These populations are very different from the Boltzmann distribution, also shown on this figure for the SiH_2_ vibrational states at *T* = 1000 K, which exhibits an exponential decay with *ν* = 0 at its maximum. It nicely demonstrates that it would not be possible to associate a single vibrational temperature for this bell-shaped distribution.

### 4.3 Non-LTE intensity simulations

A non-LTE absorption line intensity *I*_fi_ (cm per molecule) can be calculated as17
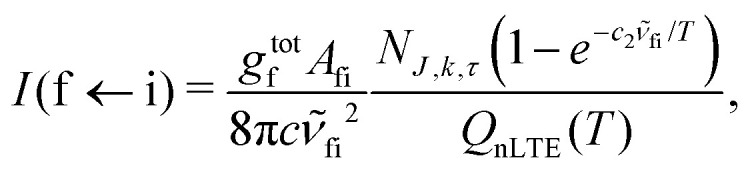
where *A*_fi_ is the Einstein *A* coefficient (*s*^−1^), *ν̃*_fi_ is the transition wavenumber (cm^−1^), *Q*_nLTE_(*T*) is the non-LTE partition function defined in eqn (3).

In order to simulate absorption spectra of the gas phase SiH_2_ assuming a non-LTE vibrational populations *N*_*ν*_1_,*ν*_2_,*ν*_3__, we use the line positions and Einstein *A* coefficients from the ExoMol CATS line list^72^ employing the ExoCross program.^92^ The ExoMol line lists are formatted as two files, a States file and a Transition file. It is described extensively elsewhere^73^ and in this paper we shall only discuss how the ExoMol format has been adapted for use in non-LTE situations.

To adapt the CATS States file^72^ for non-LTE applications an additional ‘density’ column *N*_*ν*_1_,*ν*_2_,*ν*_3__ was added as a final column, see an extract from the States file in Table 6. This column contains the weightings to the transition probabilities as populations of the vibrational levels occupied by the gas phase SiH_2_. The ‘density’ column is specific for the calculation of line lists for non-LTE molecules is not routinely included into the ExoMol States files.

**Table 6:** Extract from the modified.states file of the GM 1D non-LTE line list

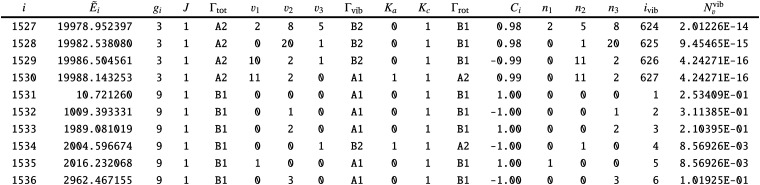

This column is read by ExoCross and used to give the population weighting to each line intensity. Evaluation of the non-LTE population *N*_*J*,*k*,*ν*_(*T*) given for each ro-vibrational state as in eqn (1) is based on the knowledge of the corresponding vibrational state (*ν*_1_,*ν*_2_,*ν*_3_) as well as the rotational energy contribution *Ẽ*^rot^_*J*,*k*_. Therefore for this approach to work it is mandatory for all ro-vibrational states to be vibrationally assigned in order to be able to subtract the vibrational contribution *Ẽ*_*v*_^vib^ from the total energy according with eqn (2). All vibrational quantum numbers (*ν*_1_,*ν*_2_,*ν*_3_) are not required, only a vibrational index indicating the vibrational state in question. In our model, all asymmetric vibrational states (*B*_2_) are not populated due to the zero overlap with the ground state of the *A*_1_ symmetry in eqn (11), as part of the completely vertical Franck–Condon approximation.

#### 4.3.1 Using the non-LTE populations from the 3D approach

**Fig. 11:**
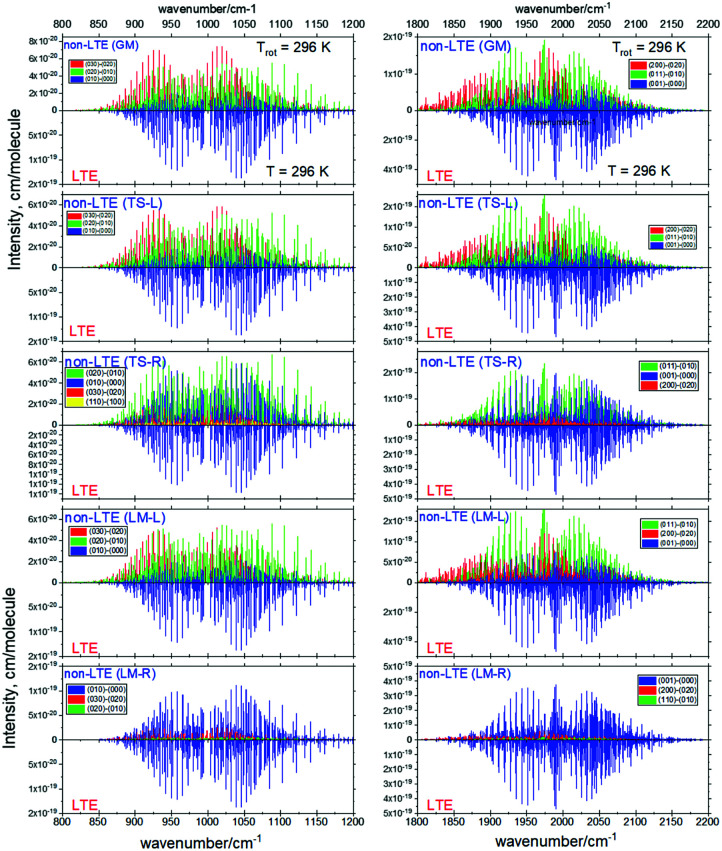
Non-LTE spectra of SiH_2_ at *T*_rot_ = 296 K corresponding to five vibrational populations GM, TS-L, TS-R, LM-L and LM-R (upper displays of each figure) and compared to the same spectra simulated using the LTE population at *T* = 296 K. The non-LTE populations were obtained using the 3D TROVE approach. Only three or four strongest bands are shown. The CATS line list was used.

Fig. 11 shows non-LTE spectra of SiH_2_ in the two main spectroscopic regions, 1000 and 2000 cm^−1^ for all five fragments considered (GM, TS-L, TS-R, LM-L and LM-R). The corresponding vibrational non-LTE populations were generated with the 3D TROVE approach. The rotational populations assume the Boltzmann distribution with the rotational temperature of *T*_rot_ = 296 K. These non-LTE spectra are compared to the corresponding LTE spectra of SiH_2_ at *T* = 296 K, which comprise mainly two fundamental bands, (0,1,0)←(0,0,0) (1000 cm^−1^ region) and (0,0,1)←(0,0,0) (2000 cm^−1^ region), with the hot bands suppressed due to the relatively low temperature. The non-LTE spectra are dominated by the hot bands (0,2,0)←(0,1,0), (0,3,0)←(0,2,0) (1000 cm^−1^ band), (1,1,0)←(0,1,0) and (1,1,0)←(0,2,0) (2000 cm^−1^ band). The centers of the hot bands are systematically red shifted compared to the fundamental band centres and serve as distinct signatures of the non-LTE effects. The *Q*-branch of (011) ← (020) is especially distinct compared to the *Q*-branch of the LTE fundamental band in the 2000 cm^−1^ region, with the difference of about 15 cm^−1^. Only the LM-R spectra of non-LTE are very similar to the LTE spectra. This is not surprising considering that the equilibrium values of *r*_SiH_ and *α*_∠H–Si–H_ of the LM-R structure are very similar to the corresponding equilibrium parameters of the gas phase SiH_2_.

The stark differences of the non-LTE spectra offer an ability for experiment to distinguish between SiH_2_ molecules produced from fragmenting Si_2_H_6_, and even to indicate dissociation channels involved.

### 4.4 *Ã*–*X̃* spectrum

The visible electronic band *Ã*–*X̃* of SiH_2_ has often been used to study different reactions involving leading to silylene.^91^ Here we used the program RENNER^117^ to simulate a non-LTE electronic spectrum of SiH_2_ with the spectroscopic model by Yurchenko *et al.*^118^ for the *Ã*^1^*B*_1_–*X̃*^1^*A*_1_ system. The model includes two empirically adjusted PESs, for the *Ã* and *X̃* states, respectively, and an *ab initio* (MRCI) *Ã*–*X̃* transition dipole moment surface (TDMS).

In the RENNER calculations, the size of the basis set originally used in Yurchenko *et al.*^118^ was reduced in order to be able to increase the rotational excitations. The main purpose of this exercise is to show a qualitative impact of the non-LTE populations on the spectral shape of the electronic band and not so much the quality of the line positions, and therefore a smaller basis set is justified. We used 18 and 12 bending basis functions for the *X̃* and *Ã* electronic states, respectively, for every |*k*| block (where |*k*| ≤ *J*). The *X̃*^1^*A*_1_ electronic state basis set included *N*_A_ = 12 stretching functions of the *A*_1_ symmetry and *N*_B_ = 10 stretching functions of the *B*_2_ symmetry. For the *Ã*^1^*B*_1_ state *N*_A_ = 10 and *N*_B_ = 8 stretching functions were used. These stretching functions were constructed from the Morse oscillator functions |*n*_1_〉|*n*_3_〉 with *n*_1_ + *n*_3_ ≤ *N*_stretch_ = 12.

A rovibronic line list for the *Ã*^1^*B*_1_–*X̃*^1^*A*_1_ of SiH_2_ was generated covering the rotational excitations up to *J*_max_ = 15 with the lower state energies (*X̃*) truncated at *hc*·25 000 cm^−1^ and the upper state energies truncated at *hc*·28 000 cm^−1^.

**Fig. 12:**
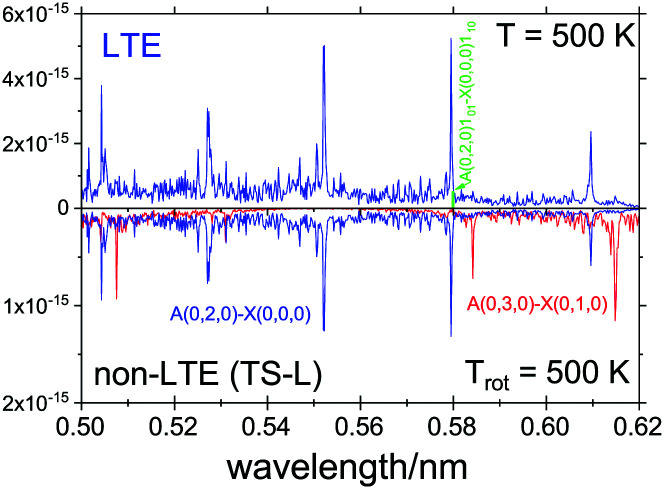
An *Ã*^1^*B*_1_–*X̃*^1^*A*_1_ spectra of SiH_2_, LTE (*T* = 500 K) and non-LTE *T*_rot_ = 500 K using the 1D model for Fragment TS-L. The energies and Einstein coefficients are generated using the RENNER program with the spectroscopic model from Yurchenko *et al.*^118^ A Gaussian line profile of HWHM = 5 cm^−1^. The popular ro-vibronic line (0,0,0)1_01_←(0,0,0)1_10_ used in measurements of reaction rates of SiH_*n*_ species^71,89,90^ is shown.

For the non-LTE simulations we used the 1D vibrational population model with the structural parameters corresponding to TS-L from Table 3. Fig. 12 shows a non-LTE electronic spectrum of SiH_2_ in the region of the band *Ã*(0,2,0)←*X̃*(0,0,0), assuming the rotational temperature *T*_rot_ = 500 K, compared to an LTE spectrum of *T* = 500 K. The non-LTE spectrum contains the hot band *Ã*(0,3,0)←*X̃*(0,1,0) which can be used to identify the non-LTE character of the system. The rovibronic line 1_01_←1_10_ belonging to this band used in a number of experimental studies involving SiH_2_ as a reaction product^71,89,90^ to estimate reaction rates. It is common for such studies to assume the Boltzmann equilibrium at different stages of the analysis of the measurements. For example, the partition function of SiH_2_ is required to estimate the number density of SiH_2_ in its lower, ground electronic state,^70,71,119^ which is directly affected by the LTE assumption. As our calculations show, the number densities of SiH_2_ as a reaction product can vary significantly under non-LTE depending on the reaction pathway and impact the experimental rates.

## 5 Conclusion

The focus of this paper is on the new features which have been added to TROVE to allow modelling the non-LTE populations of polyatomic molecules. We have demonstrated this capability by modelling the non-LTE line list of SiH_2_ calculated with 3D wavefunctions and TROVE, and compared them to non-LTE spectra of SiH_2_ modelled using a 1D harmonic approach, and the LTE line list calculated previously by ExoMol.

There are two stable isomers of disilane, a local minimum structure and a global minimum structure, with a third transition state structure also known. Non-LTE spectra of SiH_2_ corresponding to dissociation of disilane from different sides of the three disilane isomer were computed. We have shown that the non-LTE spectra of SiH_2_ are different in most cases. This is important as the spectrum of SiH_2_ is used to monitor the quantity present in a reaction as a means to track the progress of SiH_2_ + SiH_4_ → Si_2_H_6_ and Si_2_H_6_ → SiH_2_ + SiH_4_ when calculating the corresponding rate constant. If Si_2_H_6_ is decomposing at a rate slower than it is being formed, then tracking the quantity of SiH_2_ can give a rate constant that is not reflective of the speed of reaction, and merely an indication of the equilibrium balance of the two species SiH_2_ and Si_2_H_6_.

In two approaches considered, 1D and the 3D, we assume that the rotational degrees of freedom are equillibrated quickly once the dissociation from disilane occurs, hence we use the Boltzmann distribution for the rotational degrees of freedom. We also assume that the SiH_2_ fragment during the instantaneous dissociation and is fully decoupled from the rest of the Si_2_H_6_ molecule, *i.e.* can be described by a 3D wavefunction in its lowest, relaxed vibrational configuration and has the same structural parameters as the Si_2_H_6_ molecule.

We have shown that the non-LTE spectra of SiH_2_ can be calculated by the new TROVE methodology and existing ExoMol line list, and it compares well to the simpler 1D harmonic approximation published previously. The method could be applied to the non-LTE spectroscopy of other small molecules including SiH_4_, which has not been explored here.

We have also shown that despite the many approximations used in the 1D approximation (separability of the modes, Harmonic approximate *etc.*), the results compare well to the results obtained using the full 3D approach. This lends confidence in using the simplified but robust 1D approach in similar non-LTE studies, as *e.g.* we have used to model the CO non-LTE spectra,^6^ recently, which are planning to explore in the future.

The methods described here can be used model the intensity distribution of the reaction products and to ascertain from what molecule the SiH_2_ dissociated from. The equilibrium structured parameters (bond lengths and angles) can be treated as effective parameters to be adjusted to reproduce the experimental spectra.

## Conflicts of interest

There are no conflicts to declare.

## Supplementary Material

CP-023-D1CP00839K-s001
